# Benefits and Challenges of *Jatropha* Meal as Novel Biofeed for Animal Production

**DOI:** 10.3390/vetsci8090179

**Published:** 2021-09-01

**Authors:** Ehsan Oskoueian, Arshin Oskoueian, Majid Shakeri, Mohammad Faseleh Jahromi

**Affiliations:** 1 Department of Research and Development, Arka Biotechnology Corporation, Mashhad 1696700, Iran; arshin.oskoueian@gmail.com (A.O.); mfjahromi@gmail.com (M.F.J.); 2 Department of Medicine, University of Washington, Seattle, WA 98109, USA

**Keywords:** *Jatropha curcas*, functional feed additive, bioactive compounds, biological activity, novel protein sources, animal production

## Abstract

*Jatropha curcas* L. has gained importance as a source of seed oil for biodiesel production. The meal contained about 60% protein with a good balance of essential amino acids, containing various bioactive compounds, including saponins, phytic acids, trypsin inhibitors, lectins, phenolics, and flavonoids, which render it as a potential biofeed for animal production. The *Jatropha* meal demonstrated various biological activities, including antioxidant, antibacterial, and anti-inflammatory effects which enhance its property as a bio-feed. The levels of these bioactive compounds in the seeds are dependent on the genotypes. The *J. curcas* possessed different varieties which are either toxic or non-toxic according to the presence of phorbol esters. The presence of phorbol esters in the meal confirmed the toxic variety of *Jatropha* resulting in the limited application of meal as a biofeed. The *Jatropha* meal devoid of phorbol esters could be applied as a biofeed in the animal production industry, and for the toxic varieties, various techniques such as physicochemical and biological treatments have been introduced to the industry to remove the phorbol esters from *Jatropha* meal. Several studies employing various cells and animals confirmed the toxicity of the phorbol esters. The molecular mechanism of action of phorbol esters is through up-regulation of PKC-β II gene, overexpression of down-stream proto-oncogenes resulted in inflammation and oxidative stress ending by apoptotic cell death. Despite the presence of valuable bioactive compounds in the *Jatropha* meal, its nutritional application is not recommended unless the phorbol esters are completely removed.

## 1. Introduction

### Jatropha curcas Plant

*Jatropha curcas* is a tall shrub that can grow between 2 and 6 m in height. This plant begins to bear fruit after 1 year, and the full productive stage is at the age of five years. The fruits of the plant are usually 1.8 cm in length and 1.2 cm in width on average, round-shaped, and on ripening contain three black seeds. After growing for five years, its seed yield ranges from 7.5 to 12 tonnes per hectare per year. The productive life of the plant is 40–50 years [1,2].

The plant itself is very sturdy and can be an excellent candidate for re-greening of eroded zones, and for those lands that are not suitable for growing more sensitive and demanding crops [3]. *Jatropha curcas* seeds contain about 50–60% oil, which makes it a good source of oil for biodiesel production. In Malaysia, there is a growing interest to grow this plant in various provinces, such as Sabah, Selangor, and Terengganu. The plantation area in Malaysia, at the end of 2008 was estimated at 750,000 acres and was expected to increase to 1.5 million acres in 2009 and 2.15 million acres in 2010 [4]. Furthermore, *Jatropha* is available on an estimated 900.000 hectares globally, 760.000 in Asia, 120.000 in Africa, and 20.000 in Latin America (Food and Agriculture Organization of the United Nations, http://www.fao.org/news/story/en/item/44142/icode/; last accessed on 23 February 2021).

There are varieties of *Jatropha curcas* known by their origins, for instance from Nicaragua (Cape Verde and Nicaragua), Nigeria (Ife), and Mexico (Papantla) [5]. However, the different varieties contain different levels of bioactive compounds, as affected by both genotypes and geophysical conditions [6]. Makkar et al. [5] categorized the *Jatropha curcas* plant as toxic and non-toxic genotypes, where the seeds of the toxic varieties contain phorbol esters, while the non-toxic varieties do not.

The *Jatropha* meal contained about 60% protein with a good balance of essential amino acids, containing various bioactive compounds, including saponins, phytic acids, trypsin inhibitors, lectins, phenolics, and flavonoids, which render it as a potential biofeed for animal production. This review aimed to highlight the potential of *Jatropha* meal as novel biofeed, and to discuss the bioactive compounds, detoxification studies, and its possible applications as a functional feed ingredient in animal diet.

## 2. Materials and Methods

A systematic review of peer-reviewed studies was performed on the published articles from 1977 up to 2021 by ISI Web of Science (www.webofknowledge.com; last accessed on 23 February 2021), Sciencedirect (www.sciencedirect.com; last accessed on 23 February 2021), Pubmed (https://www.ncbi.nlm.nih.gov; last accessed on 23 February 2021), Wiley (www.onlinelibrary.wiley.com; last accessed on 23 February 2021), and Springer (https://link.springer.com; last accessed on 23 February 2021) publishers. The *Jatropha curcas*, plant protein sources for animal feed, bioactive compounds, biological activity, novel protein sources, animal production, phorbol ester toxicity, detoxification of Jatropha, *Jatropha* meal, and *Jatropha* meal as a soybean-replacing protein source were used as keywords for the search process. The studies were chosen according to the relevancy of the title and abstract with the objective of the present work. The selection criteria for the articles were as follow: (i): chemical and phytochemical characterization of *Jatropha curcas* seeds and meal; (ii): using animal model and animal cell model to study the toxicity of the *Jatropha curcas* seeds or meal (iii) application of *Jatropha* seed or meal as an animal feed (iv) articles published in peer-reviewed journals. At the end of searching and article selection, a total of 102 published articles were selected for the review process. The extracted information from those selected papers have been divided into the following main sections: (i) *Jatropha* meal as a biofeed and its production; (ii) bioactive compounds in *Jatropha* meal and their potential; (iii) alleviation of bioactive compounds in *Jatropha* meal; (iv) in vitro digestibility of *Jatropha* meal; (v) toxicity and biological activity.

## 3. *Jatropha* Meal a Biofeed and Its Production

### 3.1. Jatropha Meal as a Novel Biofeed

For the past decades, several feed additives, such as ionophores, antibiotics, growth promoters, synthetic antioxidants, and other chemicals, have been used to enhance feed conversion efficiency, increase livestock and aquaculture productivity and decrease pollution and environmental damage arising from the animal agriculture industry. However, recently, in some countries, the use of most of these feed additives has been banned due to the antibiotic resistance in humans caused by derivatives or residues left in animal products, which has raised concerns about product safety for consumers [7]. Hence, it is important to introduce natural feed additives containing bioactive compounds to livestock and aquaculture industries to enhance their productivity [7]. In this sense, any type of material that possesses biological activities towards the improvement of animal health and production is called biofeed.

Agriculture and food industries annually produce large amounts of waste materials, including bagasse, peels, meals, pulps, and husks. Most of these residues have nutritional value and are classified as agro-industrial by-products which are gaining importance as biofeeds. Rapeseed, cottonseed, and soybean meals are common by-products of the seed oil industry. The products have been recommended as a feed additive for livestock as it contains a high level of protein and low level of fiber [8,9]. Furthermore, it contains lignin, cellulose, amino acids, proteins, and polyphenolic compounds [10]. The available compounds contain carboxyl, hydroxyl, and methyl groups, fixed anionic and cationic moieties which have antimicrobial, anti-inflammatory, and antioxidant activities.

Gallegos-Tintore et al. [11] reported the presence of bioactive peptides in *Jatropha* meal possessing free radical scavenging, reducing power and antioxidant activities. Besides, Lihua et al. [12] showed that the leaf extract of the *Jatropha curcas* plant possesses an in vitro antibiotic effect on chicken pathogens, such as *Escherichia coli*. Moreover, a recent study has shown the anti-tick activity of *Jatropha* meal in rabbits [13,14].

### 3.2. Production Method of Jatropha Meal

After harvesting, the *Jatropha* fruits are dehusked and the seeds are sun-dried before the deshelling process. The kernels are pressed for oil extraction, resulting in a protein-rich *Jatropha* meal. The oil extraction can be carried out with two methods: mechanical pressing and solvent extraction. Mechanical extraction is more practical in the industry, but the final products are not pure and contain a significant amount of water, metals, and dust contents.

The second method of extraction is solvent extraction which has advantages over the first method in obtaining a higher yield of oil, almost 98%, especially with materials having low oil content. However, the second method does not apply to oilseeds with high oil content. When the oil extraction process is performed at a low temperature, the solvent extraction method has another advantage because it gives better quality of produced oil [6].

## 4. Bioactive Compounds in *Jatropha* Meal and Their Potential

### 4.1. Phenolics

*Phenolic* compounds are a class of chemical compounds, widely available throughout the plant species, comprising hydroxyl groups (-OH) bonded directly to an aromatic hydrocarbon group [13]. The hydroxyl groups are usually the functional moieties that determine the biological activity of each phenolic compound. Phenolics are usually found as esters or glycosides rather than as free compounds [15]. Previous research reported the presence of various phenolic compounds in *Jatropha curcas* seed, leaf, root, and stem [16,17,18]. Makkar et al. [19] reported that *Jatropha* meal had a total phenolic content ranging from 0.2 to 0.4% tannic acid equivalent and tannins at 0.02 to 0.04% tannic acid equivalent, whereas condensed tannins were not detected.

Some phenolics are considered to be powerful antioxidants, being more potent than vitamins E, C, and carotenoids [20]. Moreover, phenolic compounds are also known to have antimicrobial activity against pathogens and they act directly against microorganisms by inhibiting virulence factors. Recently, Monteiro et al. [2] evaluated the anthelmintic activity of the hexane, ethyl acetate, and ethanol extracts obtained from the seeds of *Jatropha curcas* against *Haemonchus contortus* larvae. This study demonstrated that the ethanol extract was most promising to inhibit larval exsheathment (99.8%). This activity was attributed to the presence of the total phenolic compounds and tannins with values of 108 mg/g (gallic acid equivalent) and 3.2 mg/g (tannic acid equivalent), respectively.

The response of rumen microbes to polyphenolic compounds varies. Polyphenolics have been reported to decrease the methanogens activity in the rumen [21]. Various response inhibitory activity of Sainfoin (*Onobrychis viciifolia*) tannins on the growth of *Butyrivibrio fibrisolvens*, *Ruminobacter amylophilus*, and *Streptococcus bovis* confirmed that rumen bacteria respond differently depending on species of microorganism and the type of tannin [22]. A study performed by Zuhainis et al. [23] showed that various phenolic compounds could play a major role in the reduction of the enzymes and fermentation activities of the rumen fungus *Neocallimastix frontalis* B9. In another study, Makkar et al. [24] indicated that quebracho condensed tannins at dosage of 0.1–0.4 mg/mL could decrease the number of total protozoa, entodiniomorphs, and holotrichs. However, ruminants, such as cattle, goats, and sheep are capable of digesting the compounds to reduce the negative effects on health. The majority of the microorganisms that have an important role in the biodegradation of tannins belong to the genus Streptococcus (e.g., *S. bovis*, *S. caprinus*, and *S. gallolyticus*) [25].

### 4.2. Flavonoids

Flavonoids are widely distributed in the leaves, seeds, bark, and flowers of plants. Over 4000 flavonoids have been identified to date [26]. Flavonoids are benzo-γ-pyrone derivatives consisting of phenolic and pyran rings and classified according to carbon skeleton and substitution. There are high numbers of structurally related compounds with chromane-type skeletons, with a phenyl substituent in the C2 or C3 position [27]. The most important flavonoids subclasses are as follows: flavone, flavonol, isoflavone, flavanone, chalcone, anthocyanidin, catechin, and flavanonol. The subclasses of flavonoids differ in the arrangements of hydroxyl, methoxy, and glycosidic side groups, and in the conjugation between the A- and B- rings [28]. Some of these compounds are responsible for the flower colours and act as a defensive system to protect the plant against ultraviolet radiation or pathogens and herbivores [29]. It has been shown that various flavonoid compounds are available in *Jatropha curcas* leaf, root, and stem [16,17,18]. Flavonoids are attributed to their antioxidant and chelating abilities [30], antimicrobial activities [31], anti-inflammatory, and anti-cancer properties [32].

Flavonoids in the rumen might be metabolized to more active compounds and their interaction with rumen microbes could be positive or negative [31]. Previously, Broudiscou and Lassalas [33] reported that isoquercitrin, a flavonoid present in *Equisetum arvense*, increased the amount of acetate and propionate during rumen microbial fermentation in batch culture. Recently Serra et al. [34] reported that a mixture comprising naringin and bitter orange extract could inhibit acidosis caused by *Streptococcus bovis* through the inhibition of its growth rate in the intensive fattening of ruminants. Moreover, in another study, supplementation of daidzein at a dose of 10 mg/kg of feed improved the population of rumen bacterial species in goats [35]. Flavonoids are believed to have direct effects against methanogens [36], and Tavendale et al. [37] observed the suppression of methane production in a broth culture of *Methanobrevibacter ruminantium* strain DSM 1093 when flavonol was added.

### 4.3. Saponins

Saponins are phytochemical compounds with high molecular weight glycosides available in plant genera and act as a defense system against pathogens, pests, and predators. The availability of both polar (sugar) and nonpolar (steroid or triterpene) groups provide saponins with strong surface-active properties that are responsible for many of its adverse and beneficial effects [38]. The presence of saponins in the *Jatropha* meal obtained from toxic and non-toxic varieties ranged from 2.6 to 3.4% diosgenin equivalents [5]. The observation of very similar content of saponins in the toxic and non-toxic varieties of *Jatropha curcas* suggested their nutritional safety [19].

Saponins are known for their antimicrobial, antioxidant, and cytotoxic activities [39], also recognized by their hemolytic and foaming properties. Bitter taste and astringency of plant materials could be a result of high concentrations of saponin [38]. The beneficial biological activities of saponins include hypocholesterolemic effects, the improvement of growth and production in various animal species, anthelmintic activity, antimicrobial activity, improved reproductive efficiency, and have been comprehensively discussed by Rochfort et al. [40]. The inclusion of tea saponin (10, 20, 30, and 40 g/kg substrate) to rumen fluid in the in vitro gas production tests and incubation for 24 h decreased the protozoa counts significantly by 19, 25, 45, and 79%, and a real-time PCR-based technique also confirmed the antiprotozoal effect of the total saponin fraction [41,42]. However, rumen microbes may adapt to the presence of saponins, as was observed in Ethiopian sheep, which provided a microbial population in the rumen capable of degrading plant saponins [43].

### 4.4. Phytic Acid

Phytic acid or phytate is the hexaphosphoric ester and the principal storage form of phosphorus in the plant. It has an adverse effect at a wide range of pH values, indicating a strong affinity to bind to calcium, zinc, and iron, resulting in malabsorption of minerals. Furthermore, phytate forms indigestible complex with proteins and lipids, which reduces their digestibility [38,44]. It also reduces protein digestibility by forming complexes and interacting with trypsin and pepsin [45]. Recently, phytic acid has been considered with the additional role, protective role in carcinogenesis, antioxidant properties, hypolipidaemic activity, antiplatelet activity, and to be active against diabetes mellitus, dental caries, and renal lithiasis [44].

Phytic acid in monogastric nutrition is more important than in polygastric animals since polygastric animals benefit from microbial phytase, which allows the digestion of phytate [46]. *Mitsuokella jalaludinii* and *Selenomonas ruminantium* JY35 are representatives of a unique class of phytases, which produce bacteria isolated from the rumen [47,48]. Two studies have reported the phytate content of *Jatropha curcas* (Makkar et al. [5] and Martinez-Herrera et al. [3]), which were in the range of 6 to 10%.

### 4.5. Lectins

Lectins are proteins or named phytohemagglutinin that contain covalently bound sugar moieties and are glycoproteins with the ability of binding to specific carbohydrate structural epitopes without altering their structure [49]. The primary effect of lectins is mediated through binding to the mucosa of the intestinal wall, which results, in a decreased absorption of nutrients. This effect may cause a decreased nutrient digestibility, decreased nitrogen retention, reduced weight gain, and a less efficient feed conversion [50].

One of the main property of the compound is to agglutinate the red blood cells, as a result of the interaction of multiple binding sites on the lectin molecule with specific glycoconjugate receptors on the surface of the cell membrane [38]. Furthermore, it has other functions such as lowering of insulin levels, inhibition of the proteases in the intestine, degenerating changes in some organs such as liver and kidney, increasing the endogenous loss of protein catabolism, and releasing stored fat, disturbing the mineral metabolism and absorption of non-heme iron and lipid, and failing immune systems.

*Jatropha* meal contains 51 to 102 U (U: 1/mg of meal that produced hemagglutination per ml of assay medium) for nontoxic and toxic genotypes, respectively [51], and this range of lectin content was reported to be similar to that of soybean meal [52]. Plant lectins have been widely studied for their biological and therapeutic properties. They bind selectively to cell membrane receptors and induce cytotoxicity, inhibit tumor cell growth, inhibit protein synthesis by binding to ribosomes, down-regulate telomerase activity, and reduce angiogenesis. Curcain is the lectin fraction isolated from the latex of *Jatropha curcas* and showed wound-healing properties, whereas curcin, isolated from the seed of *Jatropha curcas* appeared to have cytotoxic activity against gastric cancer, mouse myeloma, and human hepatoma cell lines with IC_50_ values of 0.15–0.32 mg/L, 0.35–0.97 mg/L, and 2.74–3.58 mg/L, respectively [51]. Baintner et al. [53] studied the binding and degradation rate of 15 plant lectins by rumen microbes and observed that the preferential binding of rumen microbes to lectin contributed to their various degradation rates. Lectins are also heat-labile and easily inactivated by heat treatment [5].

### 4.6. Trypsin Inhibitors

Trypsin inhibitors are widely available in seeds whereas they can induce pancreatic hypertrophy/hyperplasia, leading to an increase in the size of the pancreas and the secretion of digestive enzymes, including trypsin, chymotrypsin, and elastase. It causes growth depression by endogenous loss of amino acids in the form of enzymes being secreted by a hyperactive pancreas [38]. The major effects of trypsin inhibitors in monogastric farm animals are the reduction of activity of (chymo) trypsin, pancreas hypertrophy, and the increased secretion of pancreatic enzymes [54].

A study conducted by Makkar et al. [55] showed changes in trypsin inhibitor activity of *Jatropha* meal in toxic or nontoxic genotypes when it was supplemented at 18.4 to 27.3 mg/g. In another study performed by Martinez-Herrera et al. [3] showed that the amount of trypsin inhibitory activity in *Jatropha* meal increases when it was supplemented at higher doses, from 33.1 up to 36.4 mg/g. Trypsin inhibitors could be inactivated at 121 °C for a minimum of 30 min. Despite their negative nutritional effects, these compounds have positive effects such as antifungal activity, antimalarial activity, antiproliferative activity toward leukemia, lymphoma, and ovarian cancer cells, and HIV-1 reverse transcriptase inhibitory activity [56]. Carp fish (*Cyprinus carpio*) fed *Jatropha* meal containing 24.8 mg trypsin inhibitors/g did not show any remarkable difference in their growth performance, which indicated their tolerance to the presence of high levels of trypsin [57]. Ruminants are also considered to be less sensitive to plant bioactive compounds as compared to monogastric since the rumen microbes can degrade trypsin inhibitors, lectins, and phytate to a substantial extent [51].

### 4.7. Phorbol Esters

Phorbol is a member of the tigliane family of diterpenes and isolated as the hydrolysis product of croton oil. It has two hydroxyl groups on neighboring carbon atoms that are esterified by fatty acids [56]. They are very soluble in most polar organic solvents and widely distributed in plant genera in the families of *Euphorbiaceae* and *Thymelaeaceae*.

The phorbol esters mimic the action of diacylglycerol (DAG), the activator of protein kinase C (PKC), which regulates different signal transduction pathways and other cellular metabolic activities [58]. The binding of phorbol esters to PKC is the first step in the activation of PKC, and this binding is saturable and occurs through specific interactions within the C1 domain in the regulatory region of the PKC molecule [59]. There are nine PKC genes, which code for isozymes classified into three groups: classical PKCs (cPKCs: PKC-α, PKC-β I, PKC-β II, and PKC-γ), novel PKCs (nPKCs: PKC-δ, PKC-ε, PKC-η, and PKC-θ), and atypical PKCs (aPKCs: PKC-ζ and PKC-ι) [35]. Different phorbol esters may activate different PKC isozymes exerting various biological activities such as growth inhibitory responses, apoptosis, tumorogenesis, or cell differentiation [60,61,62].

In fact, the biological activities of phorbol esters are structural dependent and the presence of functional groups may determine their biological properties. Thus, different phorbol esters activate different protein kinase C (PKC) isozymes in the cells, which could trigger different pathways and may result different symptoms in animals [58]. That is why some naturally occurring phorbol esters capable of inhibiting cells proliferation [63,64,65], while others could increase cell proliferation, induce inflammation leading to the tumor formation [58,66]. For instance, phorbol 12-myristate 13-acetate present in the croton oil appeared to be a potent tumor promoter [58]. Although there have been various reports dealing with the toxicity evaluations of the *Jatropha* meal using different animal models [67].

Previous research indicated that the cellular response to phorbol esters is critically dependent on the experimental conditions as to whether the pro-apoptotic or anti-apoptotic effect is observed [68,69]. Depending on the experimental conditions, the different types of phorbol ester and its functional groups may activate different PKCs. The activated PKC-β I induces the translocation of CD11b to the cell surface and affects cell differentiation. PKC-β II, PKC-ε, and PKC-δ act through the activation of proto-oncogenes and JNK cascades, followed by TNF-α and IL-1β, and finally affecting cell differentiation. Generally, after prolonged exposure to phorbol esters, the PKC-δ is cleaved to PKM-δ which affects cytochrome c release by the mitochondria leading to apoptosis. Amemiya et al. [70] suggested the mechanism of action of phorbol esters is through activation of PKC-β, followed by triggering the expression of proto-oncogenes, including c-Fos and c-Jun. The Fos family dimerizes with c-Jun to form the AP-1 transcription factor, which upregulates the transcription of a diverse range of genes involved in many diverse effects from proliferation and differentiation, to defend against invasion and cell damage. In addition, the overexpression of c-Jun and c-Fos genes may lead to an elevation in the expression of cyclooxygenase-2 (COX2) and induction of inflammation [71]. Gomperts et al. [68] also reported the anti-proliferative and pro-apoptotic signal as a result of prolonged treatment with phorbol-12-myristate 13-acetate (PMA).

The other reported biological activities of phorbol esters include induction of structural changes in the parasite *Leishmania amazonensis* at a PMA concentration of 20 ng/mL of PMA [72], and antimycobacterial activity of the phorbols isolated from *Sapium indicum* against mycobaterium with the minimum inhibitory concentration between 3.12 and 200 μg/mL [73].

### 4.8. Jatropha Meal Phorbol Esters

Haas and Mittelbach [74] confirmed the presence of five phorbol esters in the *Jatropha curcas* seed. The five phorbol esters were identified and characterized and appear as four peaks in the high-performance liquid chromatography chromatogram [51,75]. The phorbol esters are considered to be the most biologically active compounds present in the *Jatropha* meal [51].

Recently, Saetae, and Suntornsuk [76] reported the antifungal activity of *Jatropha* meal ethanolic extract against *Fusarium oxysporum*, *Pythium aphanidermatum*, *Lasiodiplodia theobromae*, *Curvularia lunata*, *Fusarium semitectum*, *Colletotrichum capsici* and *Colletotrichum gloeosporiodes*. The phorbol esters were attributed to be the constituents mainly responsible for the antifungal activities observed in this study.

The biodegradation of the phorbol esters from *Jatropha* meal by soil microorganisms was reported by Devappa et al. [77]. The *Jatropha* meal containing 0.37 mg/g phorbol esters completely lost the phorbol esters content upon 15 days incubation in soil with 230 g/kg moisture at a temperature of 32 °C. Isolated phorbol esters at a concentration of 2.6 mg/g soil were completely degraded after 9 days in the soil containing 230 g/kg moisture at 42 °C. Increasing the temperature and the moisture content increased the rate of phorbol esters degradation.

In an early study conducted by Horiuchi and Sugimura [78], the phorbol ester factor I isolated from *Jatropha curcas* seed was shown to possess skin tumor-promoting activity; however, Devappa et al. [51] believed that the phorbol esters themselves do not induce tumors. In addition, not all phorbol esters are toxic, and their activity is strictly structure-dependent with the α form of phorbol being inactive and the β form being active [58]. Detailed information regarding the biological activity of the phorbol esters present in *Jatropha* meal has not been elucidated so far.

### 4.9. Jatropha Oil Fatty Acids Composition

Most of the available fatty acids in *Jatropha* oil are oleic acid, linoleic acid, palmitic acid, and stearic acid [79,80]. The fatty acid composition of *Jatropha curcas* and other common vegetables are presented in Table 1.

## 5. Alleviation of Bioactive Compounds in *Jatropha* Meal

### 5.1. Physical Processing

It has been reported that various types of physical processing, including roasting, moist heat treatment (autoclaving), and irradiation have been evaluated for removal of the bioactive compounds present in *Jatropha* meal [3,81]. Overall, heat treatment is necessary to inactivate the trypsin inhibitors and lectins. Adjustment of moisture of the *Jatropha* meal to 66% and heating at 121 °C for 30 min inactivated the total trypsin inhibitors and lectin content [19,57,82]. Moist heat treatment appeared to be the most effective method, compared with irradiation and roasting, in the inactivation of lectin and trypsin inhibitors. However, the content of the saponins, phorbol esters, phytic acid, and the total phenolic compounds was not affected.

### 5.2. Chemical Processing

It was reported by Aregehore et al. [82] that the varied efficiency of several treatments on the concentration of phorbol esters with sodium hydroxide (50%) and with methanol (95%). The heating treatment at 121 °C, 30 min (with 66% moisture), followed by washing four times with methanol removed the phorbol esters; however, it was not an economically viable option for commercial processing [81]. Similarly, Martinez-Herrera et al. [3] also reported the high efficiency of phorbol ester removal (98%) through a solvent extraction procedure.

Xiao et al. [83] reported that a combination of hydrolytic enzymes (cellulase plus pectinase) and washing with ethanol (65%) could completely remove the phorbol esters. The phytates, phenolic compounds, saponins, trypsin inhibitors, and lectin were decreased to acceptable levels, which are lower than those in soybean meal. Kumar et al. [84], Wang et al. [85] and Harter et al. [86] reported the full deactivation of lectin and phorbol esters by extracting with 90% methanol-sodium hydroxide, followed by steam treatment (103.4 kPa and 121 °C for 15 min). Recently, Katole et al. [87] soaked the *Jatropha* meal with sodium chloride, sodium hydroxide, and calcium hydroxide (overnight), followed by heating (100 °C for 30 min). The lectins were fully inactivated, but the phorbol esters content was reduced to 85.0, 75.0, and 83.2% using sodium chloride, sodium hydroxide, and calcium hydroxide, respectively.

### 5.3. Biological Processing

Solid-state fermentation (SSF) has been developed for the biodegradation and biodetoxification of some bioactive compounds in agro-industrial products and wastes [88]. Degradation of the *Jatropha* meal phorbol esters by *Pseudomonas aeruginosa* PseA strain during solid-state fermentation (SSF) has been reported by Joshi et al. [20], although the safety of the new compounds produced as a result of phorbol esters biodegradation have not been investigated by these researchers. In a similar study, Abdel-Hafez et al. [65] reported the potential of several isolates from the human intestine, such as *Bifidobacterium adolescentis*, *Clostridium butyricum*, *Clostridium innocuum* ES 24-06, *Peptostreptococcus anaerobius* 0240, *P. intermedius* EBF 77/25, and *Proteus mirabillis* S2 in the degradation of more than 90% of the phorbol esters purified from croton oil.

De Barros et al. [89] reported the fermentation of *Jatropha* meal with *Ganoderma resinaceum*, *Bjerkandera adusta*, and *Phlebia rufa* and observed a reduction of phorbol esters content by 20%, 91%, and 97%, respectively. Efficiency in the degradation of the phorbol ester by white-rot-fungi was attributed to the enzymatic reactions which occurred during the colonization stage.

## 6. In Vitro Digestibility of *Jatropha* Meal

The in vitro rumen digestibility of *Jatropha* meal reported for two varieties (Cape Verde and Nicaragua) and the non-toxic provenance from 24 h of gas production were found to be 78.0%, 78.0%, and 78.4% for the organic matter, and 10.9, 10.7, and 10.8 MJ/kg for the metabolizable energy, respectively [55]. Heat treatment caused Maillard reactions and was useful to suppress the in vitro rumen protein degradability of *Jatropha* meal. Aderibigbe et al. [90] observed the suppression of the in vitro digestibility of nitrogen in *Jatropha* meal upon heating at 160 °C for 120 min from 73.3% to 38.6%.

*Jatropha* meal is rich in protein but low in vitro rumen degradable protein as compared to soybean. The presence of more than 50% glutelin in the *Jatropha* meal leads to the low solubility of *Jatropha* meal protein in the rumen. Hence, a high amount of rumen undegradable protein available post-ruminally could be utilized by gastric digestion which would enhance polygastric production [91].

The phorbol esters are considered to be the most biologically active compounds present in *Jatropha* meal [51]. Despite the biodegradability [65,92] and antimicrobial activity [73,76] of the phorbol esters, Makkar and Becker [15] reported that rumen microbes do not degrade phorbol esters presented in *Jatropha* meal, and phorbol esters do not adversely affect rumen fermentation.

The effects of heat treatment in the inactivation of lectin and trypsin inhibitors are well documented. However, the saponins, phorbol esters, and phenolic compounds alleviation varied depending on the treatment methods used. The effects of *Jatropha* meal bioactive compounds and their alleviation on the improvement of rumen fermentation have not been extensively studied. Probably, the response of the rumen fermentation to the broad effects of treatments on alteration of the chemical constituent was much greater to hide the effects of bioactive compounds alleviation on rumen fermentation.

## 7. Toxicity and Biological Activity

### 7.1. Animal Trials

The effects of feeding *Jatropha* seeds containing phorbol esters have been studied extensively in different animal species, including goats, sheep, mice, rats, and fish [93]. Emerging literature shows that the direct feeding of *Jatropha* meal or processed meal shows different physiological effects in different organisms as a result of the different levels of bioactive compounds. The direct feeding of *Jatropha* meal produced mild to severe symptoms in various organs of different animals.

Feeding Nubian goats supplemented with 5 or 10 g/kg/d *Jatropha* seed at the age of 5–8 months (bodyweight 8–22 kg) showed the symptoms of toxicity and mortality. Furthermore, the animals showed other adverse effects such as lack of appetite, drinking less water led to dehydration, diarrhea, decreased glucose levels, and an increase in the serum arginase and glutamate oxaloacetate transaminase (GOT) activities. The results also revealed hemorrhage in the rumen, reticulum, kidneys, spleen, and heart, catarrhal or hemorrhagic abomasitis and enteritis, congestion and edema of the lung, and excessive fluid in serous cavities [94].

A study conducted by Ahmed and Adam [95] also reported the severe alteration in serum aspartate aminotransferase (AST) activity, total protein, serum albumin, hemoglobin, percent cell volume, and red blood cells when the animals were fed with different dosages of *Jatropha* seeds at 0.05, 0.5, and 1 g/kg/d.

Katole et al. [87] conducted a feeding trial with sheep using soybean meal (control) and *Jatropha* meal treated with sodium chloride and calcium hydroxide as protein sources. Their results showed the dry matter, organic matter, crude protein intake, and blood metabolites including packed cell volume, serum albumin, glucose, serum urea, triiodothyronine, thyroxine, testosterone, and alkaline phosphatase activity contents decreased in sheep fed *Jatropha* meal when compared to the control. The lactate dehydrogenase and aspartate aminotransferase activities also increased in these groups compared to the control. Although the phorbol ester content was markedly decreased to 0.3 mg/g DM by processing, this was not sufficient to reach a safe level for feeding sheep.

In another study, supplementing a diet with 10 g of leaves of the *Jatropha curcas* per kg body weight showed better results for dehydration and softer feces, whereas there was an increase in aspartate aminotransferase ammonia and potassium and a decrease in total protein and calcium in calves when *Jatropha curcas* seed was supplemented at a dose of 0.025 g/kg for two weeks. The calves showed signs of poisoning.

### 7.2. Animal Cell Models

Lin et al. [96] reported that *Jatropha curcas* kernel possessed antiproliferative activity against different cancer and normal cell lines. The *Jatropha curcas* phorbol esters were also reported to exert cytotoxic properties in various cancer cell model studies [51,97]. Aiyelaagbe et al. [98] reported the strong growth inhibition potential of *Jatropha curcas* root extract against L5178y mouse lymphoma and HeLa human cervix carcinoma cell lines, while they cause none or only very low activity against neuronal cells (PC12).

Different varieties of *Jatropha* contain different levels and types of bioactive compounds responsible for the various biological activities, which could determine the beneficial effects to the animal that consume them. Hence, the identification of these compounds presents in the local *Jatropha* meal and their effects have to be evaluated before its application as a biofeed is elucidated.

### 7.3. Detoxification Methods for Jatropha Meal

Ionizing radiation treatment is one of the reliable methods to inactivate certain toxic components, such as phorbol esters, phytates, saponins, and lectins. Some compounds such as protease inhibitors and lectins are possible to inactivate by moist heating, but not phorbol esters because they are heat stable. However, there is the possibility to reduce their concentration in the meal by using chemical treatments. Recently, detoxification has been carried out based on extraction of phorbol esters using organic solvents and inactivation of trypsin inhibitors and lectin by heat treatment [8].

### 7.4. Effects of Detoxified Jatropha on Animal Health

Detoxified *Jatropha curcas* kernel meal could be replaced with 50% of fishmeal protein in animal diets without compromising their body weight gain and nutrient utilization [99]. *Jatropha curcas* kernel has been fed to birds with no significant adverse effects in feed consumption and weight gain compared with the soybean diet. The pericaecal amino acid digestibility is varied from 0.48 (cystine) to 0.91 (methionine) in the birds [99,100]. In pigs, the average weight gain and feed conversion ratio were insignificantly different from the soybean diet. In addition, the serum and hematological parameters were not affected by the *Jatropha curcas* diet. Histopathological results revealed that the liver and kidney of pigs supplemented by *Jatropha curcas* exhibited normal histomorphology similar to a control diet [85]. Generally, *Jatropha curcas* can be replaced with a soybean diet for farm animals such as fish, turkey, and pig by as much as 50% [100]. The available compounds in *Jatropha* meal such as phenolic and saponins, which have antimicrobial activity against pathogens, render the *Jatropha* meal as a biofeed with the ability to modulate gut microbiota and improvement in animal health [101].

## 8. Conclusions

*Jatropha curcas* meal contained about 60% protein with a good balance of essential amino acids, containing various bioactive compounds, including saponins, phytic acids, trypsin inhibitors, lectins, phenolics, and flavonoids, which render it as a potential biofeed for animal production. The *Jatropha* meal demonstrated various biological activities, including antioxidant, antibacterial, and anti-inflammatory effects which enhances its property as a biofeed. The *Jatropha curcas* possessed different varieties which are either toxic or non-toxic according to the presence of phorbol esters. The presence of phorbol esters in the meal limited the application of the meal as a biofeed. The molecular mechanisms of the phorbol esters toxicity are through activation of the PKC-β II gene, overexpression of the proto-oncogenes resulting in inflammation and oxidative stress ending by apoptotic cell death. The *Jatropha* meal devoid of phorbol esters could be easily applied as biofeed in the animal production industry however for the toxic varieties despite the presence of valuable bioactive compounds in the *Jatropha* meal its nutritional application is not recommended unless the phorbol esters are completely removed.

## Figures and Tables

**Table 1:** Major fatty acid composition (%) of *Jatropha curcas* oil and other oils [79,80].

Fatty Acid	Sunflower	Palm Kernel	Soybean	Palm	*Jatropha curcas*
Oleic (%)	21.1	15.4	23.4	39.2	44.7
Linoleic (%)	66.2	2.4	53.2	10.1	32.8
Palmitic (%)	None	8.4	11.0	44.0	14.2
Stearic (%)	4.5	2.4	4.0	4.5	7.0

## Data Availability

Not applicable.
